# Transcranial magnetic stimulation over posterior parietal cortex modulates alerting and executive control processes in attention

**DOI:** 10.1111/ejn.15830

**Published:** 2022-10-12

**Authors:** Marij Middag‐van Spanje, Felix Duecker, Stefano Gallotto, Tom A. de Graaf, Caroline van Heugten, Alexander T. Sack, Teresa Schuhmann

**Affiliations:** ^1^ Department of Cognitive Neuroscience, Faculty of Psychology and Neuroscience Maastricht University Maastricht The Netherlands; ^2^ InteraktContour Nunspeet The Netherlands; ^3^ Maastricht Brain Imaging Center Maastricht The Netherlands; ^4^ Department of Neuropsychology and Psychopharmacology, Faculty of Psychology and Neuroscience Maastricht University Maastricht The Netherlands; ^5^ Department of Psychiatry and Neuropsychology, School for Mental Health and Neuroscience, Brain + Nerve Centre Maastricht University Medical Centre+ Maastricht The Netherlands; ^6^ Limburg Brain Injury Center Maastricht The Netherlands

**Keywords:** alerting, attention, executive control, Lateralized‐Attention Network Test (LANT), posterior parietal cortex (PPC), transcranial magnetic stimulation (TMS)

## Abstract

Attention includes three different functional components: generating and maintaining an alert state (alerting), orienting to sensory events (orienting), and resolving conflicts between alternative actions (executive control). Neuroimaging and patient studies suggest that the posterior parietal cortex (PPC) is involved in all three attention components. Transcranial magnetic stimulation (TMS) has repeatedly been applied over the PPC to study its functional role for shifts and maintenance of visuospatial attention. Most TMS‐PPC studies used only detection tasks or orienting paradigms to investigate TMS‐PPC effects on attention processes, neglecting the alerting and executive control components of attention. The objective of the present study was to investigate the role of PPC in all three functional components of attention: alerting, orienting, and executive control. To this end, we disrupted PPC with TMS (continuous theta‐burst stimulation), to modulate subsequent performance on the Lateralized‐Attention Network Test, used to assess the three attention components separately. Our results revealed hemifield‐specific effects on alerting and executive control functions, but we did not find stimulation effects on orienting performance. While this field of research and associated clinical development have been predominantly focused on orienting performance, our results suggest that parietal cortex and its modulation may affect other aspects of attention as well.

## Graphical abstract

Parietal cortex has been linked to alerting, orienting, and executive control, and the Lateralized‐Attention Network Test captures these attention processes. Right parietal TMS affected alerting and executive control, not orienting. Results emphasize functional PPC contributions to a range of attention mechanisms, and future TMS‐PPC research would benefit from a more inclusive approach.

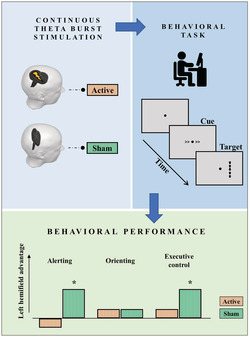

**Abbreviations**
cTBS: continuous theta‐burst stimulation
EEG: electroencephalography
LANT: Lateralized‐Attention Network Test
NIBS: noninvasive brain stimulation
PPC: posterior parietal cortex
RM ANOVA: repeated‐measures ANOVA
RT: reaction time
TMS: transcranial magnetic stimulation

## 1 INTRODUCTION

Our world consists of a large amount of stimuli and as these stimuli exceed the capacity of our brain, we have to filter the input. Attention is the cognitive process that helps us to selectively concentrate on a certain aspect of information. It is a broad concept, often defined in terms of selection, suppression and thus biasing of sensory inputs for preferred processing. The concept of attention can be divided into three different types of attention functions: alerting, orienting, and executive control (Petersen & Posner, 2012), and these functions are regulated by three relatively distinct but highly connected and partially overlapping neural networks (Fan et al., 2002, 2005, 2009; Petersen & Posner, 2012).

*Alerting* is defined as generating and maintaining a vigilant state (Coull et al., 1999; Posner & Petersen, 1990) and is responsible for spreading attention over a broad area of space and a higher alert state allows faster processing of information, independently of its spatial location. Imaging studies show that voluntarily maintaining our level of alertness over time is controlled mostly by thalamic and right frontal and parietal regions, including the posterior parietal cortex (PPC; Fan, Kolster, et al., 2007; Pardo et al., 1991; Sturm et al., 1999; Sturm et al., 2005; Sturm & Willmes, 2001). Alertness can also be modulated experimentally by presenting warning cues that indicate when, but not where, a stimulus will occur. This function is known as ‘phasic alertness’ and is associated with activity in left frontal–parietal areas and thalamus (Fan et al., 2005; Sturm & Willmes, 2001; Yanaka et al., 2010).

*Orienting* enables directional shifts of attention to a relevant spatial location (Fan et al., 2002). The influential functional‐anatomical model of Corbetta and Shulman (2011) suggests two distinct but interacting networks being responsible for spatial attentional control. On the one hand, the bilateral dorsal fronto‐parietal attention network is involved in shifts and maintenance of spatial attention and includes the PPC and frontal eye field. On the other hand, the right‐lateralized ventral fronto‐parietal attention network supports attentional re‐orienting to unexpected events and includes the temporo‐parietal junction and ventral frontal cortex (for reviews, see Corbetta & Shulman, 2011, 2002; Mesulam, 1999).

*Executive control* of attention reflects the individual's capacity to monitor and resolve conflict in the presence of competing information (Fan, Byrne, et al., 2007). Neuroimaging studies have shown activation of a network of brain areas in response to many forms of control, including task switching, inhibitory control, conflict resolution, novelty processing and error detection (for reviews, see Bush et al., 2000; Carter et al., 1999; Posner & Rothbart, 1998). The areas usually activated include the anterior cingulate cortex and supplementary motor area, the orbitofrontal cortex, the dorsolateral prefrontal cortex and portions of the basal ganglia and the thalamus (Fernandez‐Duque & Posner, 2001), but it has been hypothesized that the PPC, which is known to be involved in alerting and orienting, also plays a role in the executive control of attention (Friedman‐Hill et al., 2003; Lega et al., 2019; Marek & Dosenbach, 2018).

The abovementioned evidence for the regions and networks related to each of the three attention components, provided by brain imaging studies, is correlational in nature and limited in revealing causal relationships between task‐dependent changes in brain activity and their respective behavioural consequences. Noninvasive brain stimulation (NIBS) techniques, in particular transcranial magnetic stimulation (TMS), have become important tools in showing causality between specific brain areas and attention processes (Bien et al., 2012; Duecker & Sack, 2015a; Silvanto et al., 2009; Szczepanski & Kastner, 2013).

In healthy volunteers, TMS has repeatedly been applied over the PPC to study its functional role in visuospatial attention. TMS over PPC has been shown to affect performance on attention tasks in various experimental designs, resembling the attention deficits observed in patients with spatial hemineglect (for review, see Duecker & Sack, 2015a), but the majority of these studies used only detection tasks or spatial orienting paradigms. Thus, previous TMS‐PPC work almost exclusively addressed the ‘orienting’ component of attention, neglecting the alerting and executive control components of the framework as proposed by Petersen and Posner (2012) and particularly their potential interactions. This, in spite of the mentioned evidence from imaging studies implicating PPC in all three attention functions *and* in spite of evidence shown in behavioural studies for interactions among the three functions (Callejas et al., 2004; Callejas et al., 2005; Chica et al., 2011; Fan et al., 2009; Posner & Petersen, 1990). For instance, in studies using a tone as alerting signal, the flanker‐congruency effect (a measure of executive control) is larger on trials where an alerting signal has been previously presented, pointing to an inhibitory relationship between alerting and executive control (Callejas et al., 2005, 2004). Orienting has also shown to interact with executive control; more engagement in conflict resolution leads to an increase in benefit when one orients to the target position than when one orients to the location opposite to that of the target (Greene et al., 2008). These interactions further support the notion that the brain networks supporting the functions interact. Altogether, it is likely that TMS manipulation of PPC affects not only orienting, but also alerting and executive control.

In the current study, we were interested in the functional role of PPC in all three functional components of attention (alerting, orienting, and executive control). The Lateralized‐Attention Network Test (LANT; Asanowicz et al., 2012; Fan et al., 2002; Greene et al., 2008) is a behavioural task that simultaneously assesses the efficiency of each of the proposed functional components of attention, as well as their possible interactions.

To investigate the role of PPC in the three functions of attention, and their interactions, within each hemifield, we applied a continuous theta‐burst stimulation (cTBS) protocol (Huang et al., 2005) to the right PPC before participants performed the LANT and compared the behavioural effects to sham stimulation. Since it has been proposed that the PPC in each hemisphere biases attention toward the contralateral hemifield (Kinsbourne, 1977), we expected a reduction in alerting (Petersen & Posner), orienting (Duecker & Sack, 2015a) and executive control (Friedman‐Hill et al., 2003; Lega et al., 2019) efficiency for the left hemifield, potentially accompanied by a shift of attentional resources toward the right hemifield.

## 2 METHODS

### 2.1 Participants

Thirty‐four volunteers (20 women; mean age = 22.79 years, SD = 3.71) from Maastricht University participated in this study in return for course credits or monetary compensation. All were right‐handed according to the Edinburgh Handedness Inventory (Oldfield, 1971), had normal or corrected‐to‐normal vision and had no psychiatric or neurological history, assessed by self‐report. Participants were screened for TMS experimentation safety prior to each testing session. The research question and hypotheses remained unknown to the participants until the end of the experiment.

### 2.2 Procedure

This study presents data that were collected as part of a larger study that consisted of in total four sessions per participant. (More information on the procedure regarding the larger study can be found in the supporting information.) The data reported here reflect LANT task performance that was collected in two of those sessions; namely in a session in which (generally) inhibitory active TMS (cTBS) was applied, and in a session in which sham TMS was applied. Stimulation conditions were counterbalanced across participants. Experimental sessions were separated by at least 4 days (mean = 13 days between active and sham TMS sessions, SD = 7.1).

At the beginning of each session, participants practiced the LANT to get accustomed to the task (36 trials), during which they received feedback if they responded incorrectly or too slowly. The individual resting motor threshold was determined in the first testing session, and the same threshold value was used for the second session. A cap indicating the electrode positions of the international 10–20 electroencephalography (EEG) positioning system was used to mark the stimulation site P4 (right PPC). Then, participants were seated in front of the computer screen with the head supported by a chin rest. After calibration of the eye tracker, (active or sham) TMS was applied to the right parietal cortex, after which participants performed the LANT. EEG was recorded between TMS application and task administration (supporting information), but time from the TMS ending and the start of the task never exceeded 5 min.

### 2.3 Task and stimuli

**FIGURE 1:**
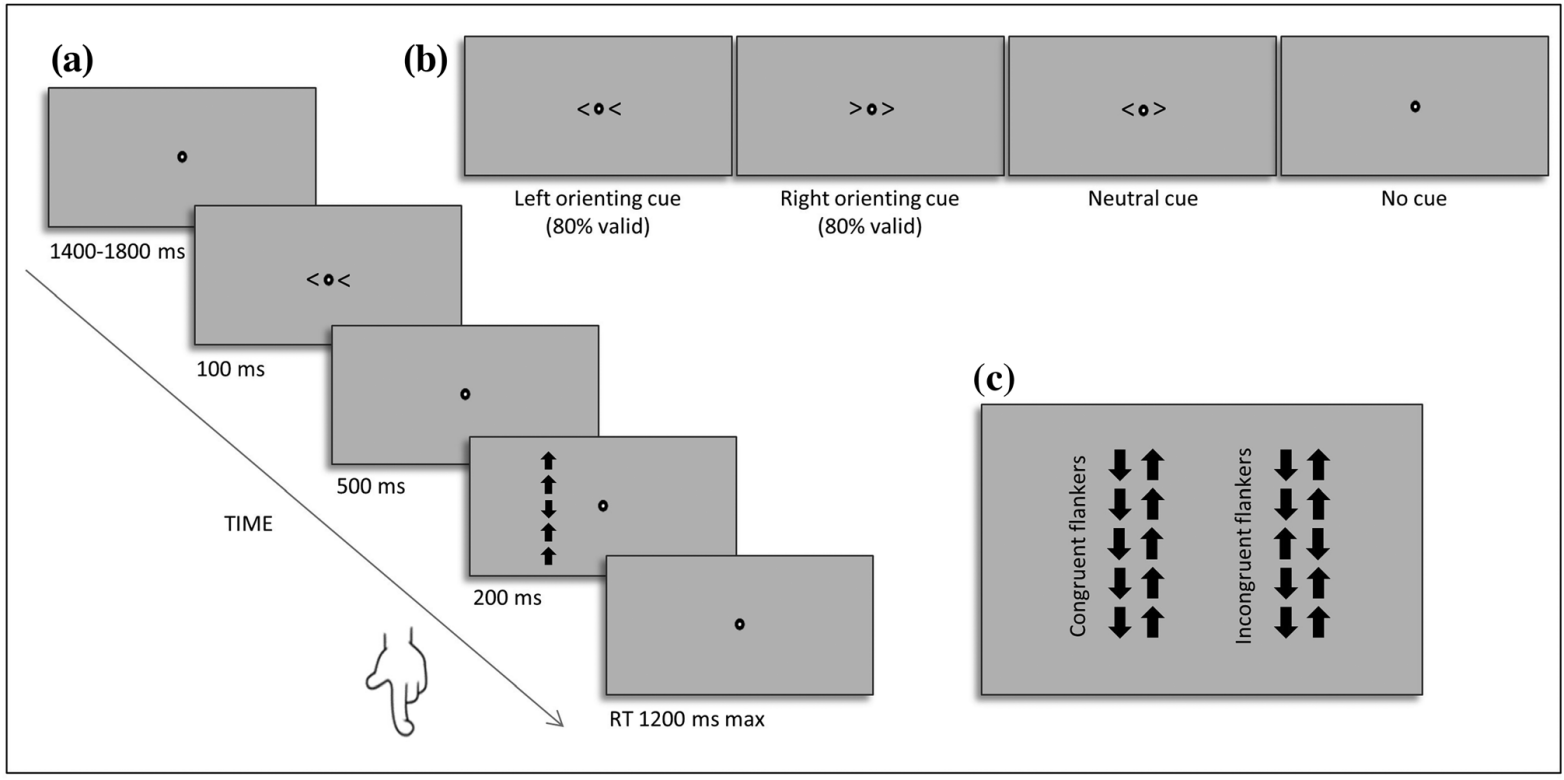
Experimental procedure for the Lateralized‐Attention Network Test. (a) An example of the sequence of events for a trial with a valid spatial cue and incongruent flankers. (b) Cue conditions (left‐orienting and right‐orienting cue, neutral cue and no cue). (c) Flanker types (congruent flankers and incongruent flankers)

Alerting, orienting, and executive control were assessed by the LANT (Figure 1; Asanowicz et al., 2012; Greene et al., 2008). In each trial, participants first focused on a fixation point, which was continuously displayed in the screen centre. After 1,400, 1,600 or 1,800 ms, a cue was presented for 100 ms. The stimulus onset asynchrony between cue and target presentation onset was 600 ms, and the main stimulus was displayed for 200 ms. The trial ended after the participant's response or, in case no response was given, after 1,200 ms.

The main stimulus comprised an array of five arrows arranged in a vertical line, presented at 7° eccentricity from the fixation point. The middle arrow was the target and pointed either up or down. The four other arrows flanked the target and pointed in a direction that was either congruent or incongruent with the target arrow. In the incongruent condition, participants had to overcome the conflict elicited by the distractor arrows. There were three cue conditions that preceded the target. A neutral cue was used to alert participants. An orienting cue was used to orient participants' attention and could be either valid (i.e., correctly indicating the location of the following target) or invalid. Therefore, the neutral (c.q. alerting) cue informed participants when the target would occur, whereas the orientingcue additionally (mis)informed them about the target location. A no‐cue reference condition was also included. Central symbolic cues were used to prompt voluntary shifts of attention (Figure 1), although it should be noted that there is evidence that arrows can orient attention involuntarily to the location they are pointing at (Hommel et al., 2001) and therefore are not purely endogenous.

The target was displayed with an equal probability on the left or right side of the screen. This presentation to one or the other hemifield enabled lateralization effects to be measured. Participants were instructed to maintain central fixation throughout the task and to indicate the direction of the target arrow as quickly and accurately as possible by pressing the up or down key on a standard keyboard with the right middle finger or index finger, respectively. Speed and accuracy of responses were measured. Participants received no feedback on their accuracy, except when they responded too slowly (RT > 1,000 ms). This measure was taken in order to keep participants vigilant to the task.

The LANT consisted of 720 trials divided into five blocks of 144 trials each, presented in randomized order. On 400 trials, the target was preceded by an orienting cue that indicated the target's location with a probability of 80%. Thus, on 320 trials the orienting cue validly predicted the target location, while on 80 trials the orienting cue was misleading. The remaining 320 trials were evenly divided into neutral‐cue (160) and no‐cue (160) trials. Concerning the flanker arrows, on one half of the trials (360), the target was flanked by congruent flankers, and on the other half by incongruent flankers. We added four warm‐up trials at the beginning of each block that were not considered in the analysis. Including short breaks between blocks, the total duration of the task was 35–40 min.

Stimuli were presented using the Presentation software package (NeuroBehavioural Systems, Albany, CA) on a Iiyama ProLite B2483HS monitor at 70 cm viewing distance. The video mode was 1,920 × 1,080 at 60 Hz, and background luminance was 100 cd/m2.

### 2.4 TMS protocol

TMS was applied with a MagPro R30 stimulator (MagVenture A/S, Farum, Denmark) and a figure‐of‐eight TMS coil (MC‐B70; inner radius = 10 mm, outer radius = 50 mm). Pulses were biphasic, with an anterior–posterior followed by posterior–anterior current direction in the brain. The coil was placed tangentially to the scalp over the electrode position P4 (based on the international 10–20 system) with the handle in posterior direction orienting 45° away from the midline. The cTBS protocol consists of a total of 600 stimuli applied in bursts of three stimuli at 20 ms intervals (50 Hz), with bursts repeated at 200 ms intervals (5 Hz) (Huang et al., 2005). Stimuli were given at an intensity of 100% of the individual resting motor threshold (mean stimulation intensity = 33.9% of maximum stimulator output, SD = 5.3, 46.8 A/μs). Resting motor threshold was determined using single pulse TMS over the right motor cortex. It was defined as the lowest intensity that elicited an observable muscle twitch of the left index finger on three of six trials (Pridmore et al., 1998; Varnava et al., 2011).

During sham stimulation, the coil was held at 90° to the participant's skull, so that no pulses perturbed underlying cortex (Hilgetag et al., 2001).

### 2.5 Eye movement control

We performed video‐based monocular eye tracking (EyeLink 1000 system, SR Research, Mississauga, Canada) to track gaze position of the participant's right eye at a sampling rate of 1,000 Hz and with high sensitivity for automatic detection. The 5‐point (centre, top, bottom, left and right) calibration and validation procedure was used, while the participant's head was supported by a chin rest. The software automatically detected eye movements and blinks when the participant performed the task. This information later allowed us to discard all trials that were contaminated by eye movements (exceeding 2° of visual angle) or blinks (M = 7.1% of trials across conditions, SD = 6.4). The critical time window ranged from 100 ms before appearance of the cue until stimulus onset. This ensured that the participant did not overtly shift attention toward the target but merely performed covert shifts of spatial attention and that behavioural effects were not to be distorted by interruptions of central fixation.

### 2.6 Data analysis

We first inspected the individual data sets of sessions with sham TMS to detect strongly deviating performances in the absence of possible stimulation effects. One participant showed accuracies around chance level in all sham conditions, so we excluded this data set from further analyses.

Besides excluding trials contaminated by eye blinks or eye movements, we also excluded trials in case of incorrect responses or misses. For each condition, trials were identified as outliers if the participant's reaction time (RT) deviated by more than 1.5 times the interquartile range (IQR) from Q1 and Q3. After application of these exclusion criteria, 84% of all trials remained for further analysis. We computed mean RTs for each condition, and the average amount of trials per smallest cell (invalid trials) was 17 trials (SD = 2.5). The average amount of trials in the other conditions was 67 (SD = 9.7) in the valid trials, 33 (SD = 5.1) in the neutral trials and 33.6 (SD = 4.9) in the no‐cue trials.

After computing mean RTs and scores of the three functions of attention (see formulae below), as a final pre‐analysis step, we inspected the RTs and scores in sessions with sham TMS. We decided to exclude one more data set from further analyses due to extreme outliers in mean RTs and scores (>3.0*IQR from Q1 and Q3) in multiple sham conditions, reducing the sample size to 32 participants.

To calculate scores of the attention functions, we conducted three separate subtractions using mean RTs of trials in which participants responded correctly (Fan et al., 2002):

1. Alerting score = no cue − neutral cue
2. Orienting score = invalid cue − valid cue
3. Executive control score = incongruent flanker − congruent flanker

We divided our analyses in two parts:

1. *LANT performance in the sham condition*. We first focused only on task performance under baseline conditions (sham stimulation) to test whether experimental manipulation of attention was successful and whether we could observe interactions among the three attention functions that previous behaviour studies have found. To this end, we submitted mean RTs of the sham condition to a repeated‐measures ANOVA (RM ANOVA) with hemifield (left and right), cue (valid, neutral, invalid and no cue) and congruency (congruent and incongruent) as within‐subject factors. Also, we submitted the alerting, orienting, and executive control scores of the baseline conditions to three RM ANOVAs, one for each score. For the ANOVAs on alerting and orienting scores, congruency was included as a within‐subject factor, and for the ANOVA on executive scores, cue was included as a within‐subject factor. By doing so, we considered interactions between the three components.
2. *Stimulation effects*. Second, we performed analyses to evaluate the differential effects of TMS‐PPC on alerting, orienting, and executive control. We chose *not* to analyse mean RTs here anymore (as we extensively did for the sham data, allowing us to discuss and compare our results with observations of previous behaviour studies) but to reduce the task conditions and *only* analyse the three scores (alerting, orienting, and executive control). Thus, to evaluate the effects of TMS, we submitted each of the three scores to a RM ANOVA. We considered interactions between the three components by including congruency and cue as within‐subject factors for the ANOVAs on alerting and orienting, and executive control, respectively.

All analyses were performed using IBM SPSS Statistics Version 25. For all RM ANOVAs, we reported the multivariate test statistics (Pillai's trace). Follow‐up analyses were conducted with paired *t* tests and Wilcoxon signed‐rank tests were performed when data were not normally distributed (according to the Shapiro–Wilk test). When Wilcoxon signed‐rank tests were performed, we reported *z* values. We used a significance level of *p* < .05.

## 3 RESULTS

Overall accuracy in the sham and the active TMS condition yielded 93.05% (SD = 9.94) and 93.55% (SD = 9.76), respectively. Mean RTs of all conditions are given in Table 1.

**TABLE 1:** Mean reaction times (RTs, in ms) of correct responses and standard error (in brackets) for each experimental condition, for sham and active TMS conditions

		Left hemifield		Right hemifield	
		Sham	Active	Sham	Active
Valid	Congruent	471.7 (10.0)	469.9 (8.3)	466.4 (10.5)	466.8 (8.6)
	Incongruent	521.0 (10.2)	529.3 (9.1)	530.3 (12.7)	531.3 (10.4)
Neutral	Congruent	473.2 (9.7)	473.9 (8.7)	470.0 (10.6)	466.7 (8.9)
	Incongruent	528.7 (10.5)	533.7 (9.0)	538.8 (13.5)	528.7 (10.2)
Invalid	Congruent	490.0 (11.0)	487.9 (8.9)	482.0 (12.3)	483.4 (10.4)
	Incongruent	539.6 (10.9)	546.1 (10.0)	547.4 (13.6)	544.1 (11.6)
No cue	Congruent	511.4 (10.1)	514.6 (8.9)	505.9 (11.3)	501.353 (8.9)
	Incongruent	563.6 (10.3)	562.7 (8.6)	562.3 (13.8)	559.4 (11.3)

### 3.1 LANT performance in the sham condition

We first focused on task performance under baseline conditions (sham stimulation). A RM ANOVA on mean RTs was performed with hemifield (left and right), cue (valid, neutral, invalid and no cue) and congruency (congruent and incongruent) as within‐subject factors. RTs differed between cue conditions (*F*[3, 29] = 63.034, *p* < .001). Subsequently, we performed planned comparisons between the cue conditions. As expected, participants responded significantly faster in valid‐cue as compared to neutral‐cue trials (*t*[31] = 3.018, *p* = .005), neutral‐cue as compared to invalid‐cue trials (*z* = 3.927, *p* < .001, *r* = .491), and invalid‐cue as compared to no‐cue trials (*t*[31] = 7.656, *p* < .001). Also, to ensure that the alerting and orienting attention components we aimed to modulate with TMS were present in a normal (baseline/sham) condition, we performed two additional *t* tests. Participants reacted significantly faster in neutral‐cue compared to no‐cue trials, resulting in a significant score of alerting (*t*[31] = 12.368, *p* < .001), and in valid‐cue compared to invalid‐cue trials, resulting in a significant score of orienting (*z* = 4.245, *p* < .001, *r* = .531). We also found a main effect of congruency (*F*[1, 31] = 223.017, *p* < .001), demonstrating significantly faster performance for congruent‐flanker than for incongruent‐flanker trials, resulting in a significant score of executive control. Both the observed cue and congruency effects supported our expected response patterns. No main effect of hemifield was found (*F*[1, 31] = .018, *p* = .894).

**FIGURE 2:**
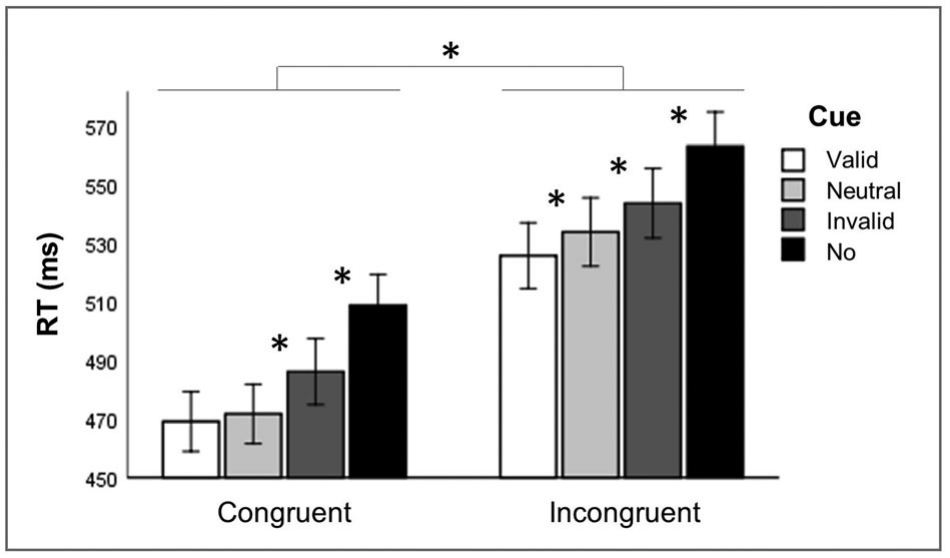
Reaction times (RT, in ms) per type of cue and congruency during sham stimulation, averaged over hemifields. Participants reacted significantly faster in neutral‐cue compared to no‐cue trials, in valid‐cue compared to invalid‐cue trials, and in congruent‐flanker compared to incongruent‐flanker trials. Thus, we efficiently measured the scores of alerting, orienting, and executive control, respectively. Asterisks (*) depict significant difference (*p* < .05). Error bars depict one standard error

There was also a significant interaction between hemifield and congruency (*F*[1, 31] = 7.630, *p* = .010). Follow‐up *t* tests showed that this interaction reflected a right hemifield advantage in congruent trials (*t*[31] = 1.951, *p* = .060), and a left hemifield advantage in incongruent trials (*t*[31] = 1.148, *p* = .260), both not significant. RTs in congruent trials were significantly faster than RTs in incongruent trials in both hemifields (left: *t*[31] = 12.218, *p* < .001; right: *t*[31] = 13.812, *p* < .001). Lastly, a significant interaction between cue and congruency (*F*[3, 29] = 2.929, *p* = .050) was found (Figure 2), so we conducted additional ANOVAs separately for alerting scores (no‐cue minus neutral‐cue trials), orienting scores (invalid‐cue minus valid‐cue trials) and executive scores (incongruent‐flanker minus congruent‐flanker trials) to test for possible relationships between the three functions of attention. These additional ANOVAs are reported in the three paragraphs below (‘Alerting’, ‘Orienting’ and ‘Executive control’). Please note that these also still concern the baseline conditions (sham stimulation) only. No significant interactions were found between hemifield and cue (*F*[3, 29] = 2.184, *p* = .111) nor between hemifield, cue and congruency (*F*[3, 29] = 1.149, *p* = .346).

#### 3.1.1 Alerting

A RM ANOVA on alerting scores was performed with hemifield (left and right) and congruency (congruent and incongruent) as within‐subject factors. This showed a significant main effect of hemifield, with lower alerting scores in the right hemifield (*F*[1, 31] = 5.622, *p* = .024), and a significant main effect of congruency, with lower alerting scores in incongruent trials (*F*[1, 31] = 4.607, *p* = .040). The latter implies an interaction between alerting and executive control. No significant interaction between hemifield and congruency was found (*F*[1, 31] = 1.949, *p* = .173).

#### 3.1.2 Orienting

A RM ANOVA on orienting scores was performed with hemifield (left and right) and congruency (congruent and incongruent) as within‐subject factors. No significant main effects of hemifield (*F*[1, 31] = .266, *p* = .610) and congruency (*F*[1, 31] = .046, *p* = .831) were found, nor an interaction between these factors (*F*[1, 31] = .029, *p* = .865).

#### 3.1.3 Executive control

We performed a RM ANOVA on executive scores with hemifield (left and right) and cue (valid, neutral, invalid and no cue) as within‐subject factors. This gave a significant main effect of hemifield, with lower executive scores (c.q. lower cost of conflict) in the left hemifield (*F*[1, 31] = 7.630, *p* = .010), and a significant main effect of cue (*F*[3, 29] = 2.929, *p* = .050). Follow‐up *t* tests revealed significantly lower executive scores for valid‐cue compared to neutral‐cue trials (*t*[31] = 2.602, *p* = .014). Executive scores did not differ between neutral‐cue and invalid‐cue trials (*t*[31] = 1.015, *p* = .318) nor between invalid‐cue and no‐cue trials (*t*[31] = .638, *p* = .528). Noteworthy, significantly higher executive scores were found for neutral‐cue trials compared to no‐cue trials (*t*[31] = 2.146, *p* = .040), which again shows the interaction between alerting and executive control processes. Executive scores did not differ between valid‐cue and invalid‐cue trials (thus no interaction between orienting and executive control, *t*[31] = .215, *p* = .831). No significant interaction between hemifield and cue was found (*F*[3, 29] = 1.149, *p* = .346).

### 3.2 Stimulation effects

We then compared active TMS to sham TMS (as control condition) to systematically evaluate the effects of stimulation on alerting, orienting, and executive control scores. To this end, we submitted each of the scores to a RM ANOVA, with hemifield included as a within‐subject factor in every analysis.

#### 3.2.1 Effects on alerting

**FIGURE 3:**
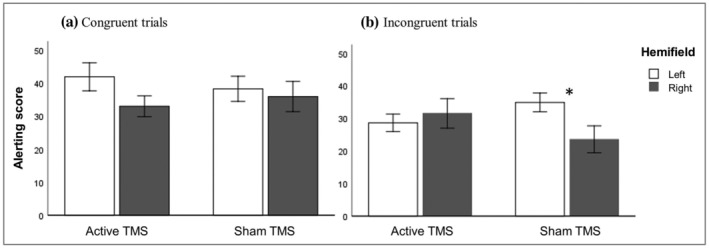
Latency estimates of alerting for congruent‐flanker and incongruent‐flanker trials. A significant three‐way interaction between stimulation, hemifield and congruency was found for the alerting effect. (a) In the congruent condition, no significant main effects or interactions were found. (b) In the incongruent condition, stimulation interacted with hemifield (significant stimulation*hemifield interaction). There was a left hemifield advantage in sham TMS (significant *p* value left vs. right in sham), but not in active TMS. Also, comparing active TMS versus sham TMS: There was a decrease of alerting efficiency in the left hemifield and an increase of alerting efficiency in the right hemifield (both comparisons not significant). Asterisks (*) depict significant difference (*p* < .05). Error bars depict one standard error

TMS effects on alerting were analysed using a RM ANOVA on alerting scores with stimulation (active and sham), hemifield (left and right) and congruency (congruent and incongruent) as within‐subject factors. The analysis revealed significant main effects of hemifield, with lower alerting scores in the right hemifield (*F*[1, 31] = 4.356, *p* = .045), and congruency, with lower scores in the incongruent trials (*F*[1, 31] = 10.098, *p* = .003). Although no main effect of stimulation was found (*F*[1, 31] = .080, *p* = .780), nor any two‐way interactions between the factors (all *p* values of >.285), crucially, there was a significant three‐way interaction between stimulation, hemifield, and congruency (*F*[1, 31] = 6.736, *p* = .014). We then analysed the alerting scores separately for the congruent and incongruent trials. In the congruent condition, there were no significant main effects (stimulation: *F*[1, 31] = .011, *p* = .918; hemifield: *F*[1, 31] = 2.967, *p* = .095) nor an interaction between stimulation and hemifield (*F*[1, 31] = 1.228, p = .276; Figure 3a). In the incongruent condition, also, no main effects were found (stimulation: *F*[1, 31] = .078, *p* = .781; hemifield: *F*[1, 31] = 1.061, *p* = .311), but importantly, the interaction between stimulation and hemifield remained significant (*F*[1, 31] = 9.178, *p* = .005; Figure 3b). Follow‐up *t* tests revealed that there was a left hemifield advantage in sham TMS but not in active TMS (left versus right in sham: *t*(31) = 2,454, *p* = .020; left versus right in active: *t*[31] = .603, *p* = .551). Also, alerting scores did not differ in active TMS compared to sham TMS, for neither hemifields (left: *t*[31] = 1.827, *p* = .077; right: *t*[31] = 1.910, *p* = .065).

#### 3.2.2 Effects on orienting

**FIGURE 4:**
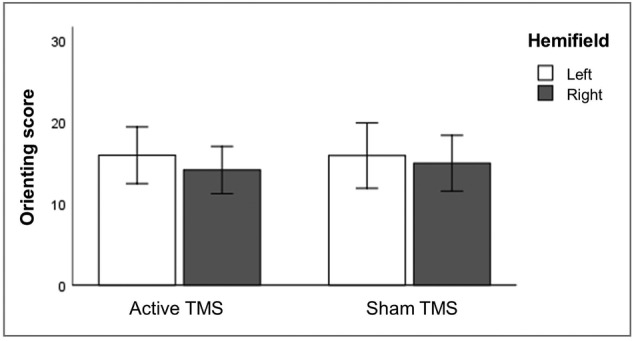
Latency estimates of orienting, averaged over congruent and incongruent trials. No significant main effects or interactions were found. Error bars depict one standard error

TMS effects on orienting were analysed using a RM ANOVA on orienting scores with stimulation (active and sham), hemifield (left and right) and congruency (congruent and incongruent) as within‐subject factors. The statistics revealed no significant main effects (stimulation: *F*[1, 31] = .062, *p* = .805; hemifield: *F*[1, 31] = .714, *p* = .405; congruency: *F*[1, 31] = .013, *p* = .909) nor interactions (all *p* values of >.628) (Figure 4).

#### 3.2.3 Effects on executive control

**FIGURE 5:**
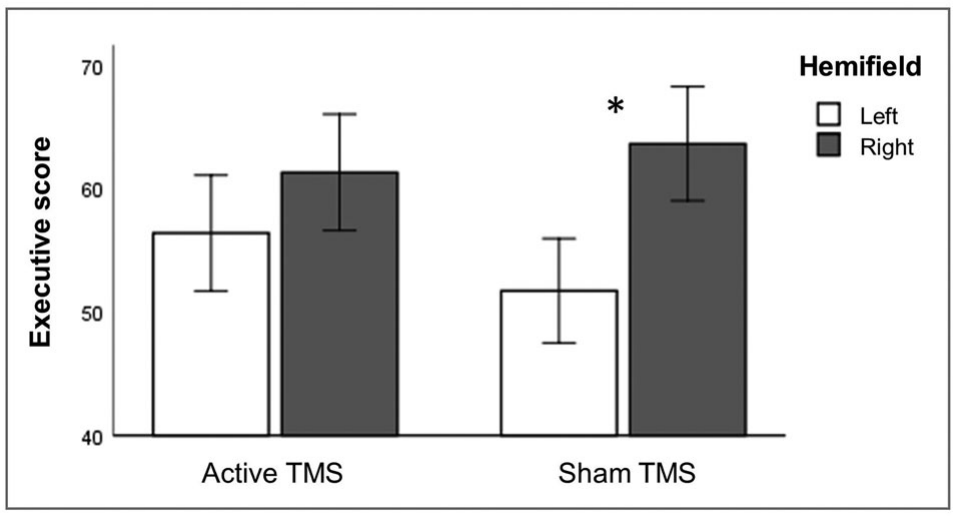
Latency estimates of executive control, averaged over all cue levels. A significant interaction between stimulation and hemifield was found for the executive control effect. There was a significant difference between the left and the right hemifield in sham TMS (significantly lower executive score for left compared to right hemifield in sham TMS) but not in active TMS. Also, comparing active TMS versus sham TMS: We found a reduced executive control efficiency in the left hemifield (i.e., higher cost of conflict, not significant) and an improved efficiency of resolving conflict processes in the right hemifield (i.e., lower cost of conflict, not significant). Asterisks (*) depict significant difference (*p* < .05). Error bars depict one standard error

TMS effects on executive control were analysed using a RM ANOVA on executive scores with stimulation (active and sham), hemifield (left and right) and cue (valid, neutral, invalid and no cue) as within‐subject factors. The analysis revealed a significant main effect of hemifield, with generally lower cost of conflict in the left hemifield (*F*[1, 31] = 4.165, *p* = .050), and a significant main effect of cue (*F*[3, 29] = 4.200, *p* = .014), due to generally higher cost of conflict in neutral‐cue compared to no‐cue trials (*p* = .003). No main effect of stimulation was found (F[1, 31] = .056, *p* = .815), but critically, there was a significant interaction between stimulation and hemifield (*F*[1, 31] = 4.188, *p* = .049; Figure 5). Follow‐up *t* tests revealed that there was a significantly lower cost of conflict in the left compared to the right hemifield in sham TMS but not in active TMS (left vs. right in sham: *t*[31] = 2.762, *p* = .010; left vs. right in active: *t*[31] = 1.102, *p* = .279). Follow‐up *t* tests also showed that executive scores did not differ in active TMS compared to sham TMS, for neither hemifields (left: *z* = 1.459, *p* = 0.145, *r* = 0.182; right: *t*[31] = .735, *p* = .468). Other interactions were non‐significant (all *p* values of >.108).

#### 3.2.4 Effects on the alerting‐executive control interaction

Although stimulation did not interact with cue in the previous section (‘Effects on executive control’), we were interested to find out whether TMS affected the alerting—executive control interaction since the executive control score depended on cue type (neutral vs. no cue) in the sham condition. To this end, we used a RM ANOVA on executive scores with stimulation (active and sham), hemifield (left and right) and cue (neutral and no cue) as within‐subject factors. We found a significant main effect of cue, with higher cost of conflict in neutral‐cue trials (*F*[1, 31] = 10.098, *p* = .003). No significant other main effects (stimulation: *F*[1, 31] = .432, *p* = .516; hemifield: *F*[1, 31] = 2.891, *p* = .099) nor two‐way interactions were found (all *p* values of >.496), but critically, there was a significant interaction between stimulation, hemifield and cue (*F*[1, 31] = 6.736, *p* = .014).

**FIGURE 6:**
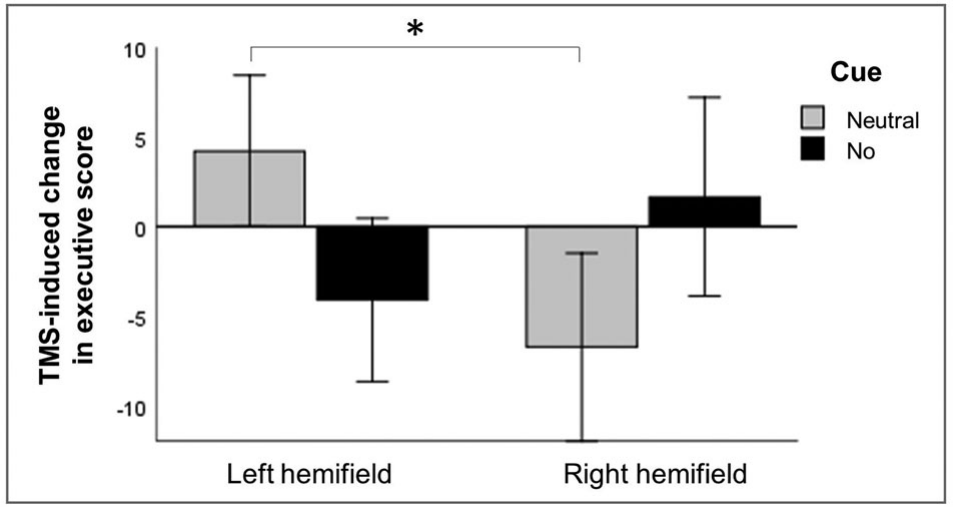
TMS‐induced change in executive score, shown separately for neutral‐cue and no‐cue trials. A higher executive score reflects a higher cost of conflict in the active TMS condition compared to the sham TMS condition. A non‐significant two‐way interaction between hemifield and cue was found. Error bars depict one standard error

To further investigate this significant three‐way interaction, we reduced the conditions by subtracting the executive scores of the sham TMS condition from the active TMS condition, giving us the stimulation‐induced changes in the executive score as dependent variable (Figure 6). We used a RM ANOVA on this new measure with hemifield (left and right) and cue (neutral and no cue) as within‐subject factors. We found no significant main effects (hemifield: *F*[1, 31] = .302, *p* = .586; cue: *F*[1, 31] < .001, *p* = .995), nor a significant interaction between hemifield and cue (*F*[1, 31] = 3.994, *p* = .055; Figure 6).

## 4 DISCUSSION

The objective of the present study was to investigate the role of PPC in the three functional components of attention as proposed by the framework of Petersen and Posner (2012; alerting, orienting, and executive control) in both hemifields. To this end, we applied a cTBS protocol to disrupt right PPC in 32 healthy volunteers and subsequently used the LANT to assess behavioural performance.

### 4.1 LANT performance in the sham condition

The behavioural outcomes under baseline conditions showed that we successfully replicated previously reported effects of cues and flanker arrows on RTs in our implementation of the LANT. We observed the typical pattern of RTs across cueing and flanker conditions; participants responded faster to targets as cues became increasingly informative, and responded slower to targets that created conflict.

Similar to Greene et al. (2008), we found no significant main effect of hemifield. However, we did find a significant interaction between hemifield and congruency. When hemifield differences were analysed separately for the congruent‐flanker and the incongruent‐flanker trials, the asymmetry turned out to be near significant in the congruent trials only. Responses were faster on right than on left congruent trials. This finding may be explained by the Simon effect because participants had to respond with their right hand. The Simon effect is the well‐known phenomenon that people are faster when responding to stimuli that are in the same relative location as the response, even though the location information is irrelevant to the actual task (Simon & Rudell, 1967). In the incongruent trials, however, we saw faster responses on left compared to right trials, although this difference was not significant. Asanowicz et al. (2012) also found a left hemifield advantage in the incongruent‐flanker trials and suggest this may indicate the right hemisphere's dominance in resolution of conflict.

We also found an interaction between cue and congruency. Significantly faster responses were observed when cued in the correct direction than responses to neutral cues, but this finding was only found in trials that created conflict (incongruent trials). In congruent trials, responses to validly cued trials were not significantly faster than responses to neutral cues (Figure 2). This is in line with the findings of Greene et al. (2008), who also found a higher facilitative effect of valid cues in incongruent trials as compared to congruent trials. Further, we corroborated previous evidence (Asanowicz et al., 2012; Callejas et al., 2005, 2004; Chica et al., 2011; Lupiáñez & Funes, 2005), by showing that valid orienting cues improve resolution of conflict (lower cost of conflict in valid‐cue trials as compared to neutral‐cue trials). This indicates that when attention is oriented to the target location there is a reduced interference from incongruent flankers.

The current study brought interesting findings on the relationship between alerting and executive control. In line with the finding of Greene et al. (2008), the alerting effect was less in incongruent trials. This suggests that the longer time that is needed to respond to targets in incongruent (more difficult) trials, cancels out the advantage of having been alerted by an alerting (c.q. neutral) cue. Or, in other words, how Greene et al. (2008, p.30) put it: ‘the more one is engaged in conflict resolution processing, the less benefit will be gained from a temporally alerting cue’. In accordance with previous studies (Callejas et al., 2005, 2004), we found a higher cost of conflict in neutral‐cue trials as compared to no‐cue trials, demonstrating an inhibitory relationship between alerting and executive control processes. This inhibitory influence between alerting and executive control has previously been described by Posner (1994). Posner proposed that the anterior cingulate cortex, which has shown to be associated with the executive control network, is inhibited when the alerting network is highly activated, to prevent the system from engaging in higher level processing in order to promote a fast response to the stimulus rather than concentrating on control functions.

To conclude, our findings on LANT performance under baseline conditions are in agreement with previous work and provide evidence on the behavioural level that different aspects of attention interact. In the following section we discuss how stimulation affected the performance of each of the three attention functions and their interactions.

### 4.2 Stimulation effects

Until now, to our knowledge only two previous studies have investigated effects of repetitive TMS protocols on the performance of all three attention functions, thereby giving a broader perspective on attention by also quantifying alerting and executive control functioning, next to the classical effects on orienting (Xu et al., 2013, 2016). However, these studies did not take hemifield‐specific effects into consideration, presenting targets above and below fixation. This limitation is particularly relevant here, because attentional biases are generally hemifield‐specific and NIBS to attention‐related regions in a single hemisphere have repeatedly been shown to have hemifield‐specific effects on task performance (Duecker et al., 2017; Duecker & Sack, 2015a). Thus, for studies that implement a hemisphere‐specific neuromodulation approach, hemispheric contributions are elementary and outcome measures should aim to capture lateralization of attention processes.

In the current study, we compared active TMS to sham TMS to investigate the role of PPC in the three functions of attention (alerting, orienting, and executive control), and their interactions, within each hemifield. Since it has been proposed that the PPC in each hemisphere biases attention toward the contralateral hemifield, we expected hemifield‐specific effects after applying TMS over right PPC, and more specifically, a rightward shift of attention. The pattern of effects we found on alerting and executive control seems to be in line with this expectation, but the effect on orienting is in contrast to previous TMS studies that have used Posner, line bisection, or extinction paradigms (Bien et al., 2012; Brighina et al., 2002; Cazzoli et al., 2009; Dambeck et al., 2006; Fierro et al., 2000; Hilgetag et al., 2001; Koch et al., 2005; Szczepanski & Kastner, 2013; Thut et al., 2005).

#### 4.2.1 Effects on alerting

Alerting was defined as performance in the no‐cue condition minus performance in the neutral‐cue condition. We found a significant interaction between brain stimulation and hemifield on incongruent trials. Follow‐up analyses showed a significant left hemifield advantage in sham TMS but not in active TMS. Compared to sham, active TMS reduced alerting performance in the left hemifield and enhanced performance in the right hemifield (note that these are interpretations based on the descriptives following the significant interaction term, rather than on the pairwise follow‐up comparisons). Thus, by applying TMS over right PPC, we found the expected rightward shift of alerting attention.

Previous studies have suggested that the alerting system is controlled mostly by right frontal and right parietal lobes (Fan et al., 2002; see also references in Introduction). Indeed, in our study, the left hemifield advantage in the sham condition indicates a right hemisphere dominance, and, in their LANT study, Greene et al. (2008) too suggest that alerting is dominated by the right hemisphere. However, Asanowicz et al. (2012) did not find a visual field asymmetry for the alerting effect and they give several interesting interpretations of this lack of asymmetry which we believe can be tested with TMS. For instance, fMRI studies have reported a greater involvement of the *left* hemisphere in the processing of alerting cues (Coull et al., 2000; Coull et al., 2001; Fan et al., 2005), and several authors suggest that this discrepancy may result from differential specialization of the hemispheres, namely, more engagement of the left hemisphere in phasic alertness, and superiority of the right hemisphere in tonic alertness (Coull et al., 2000; Okubo & Nicholls, 2008; Posner, 2008). To shed more light on the organization and laterality of the alerting network, it seems promising to further investigate the effects on alerting functioning in left and right hemifields by applying TMS over left PPC and to compare this with right PPC stimulation.

#### 4.2.2 Effects on orienting

We found no stimulation effects on orienting, which was defined as performance in the invalid‐cue condition minus performance in the valid‐cue condition. This is in contrast to several previous studies investigating spatial orienting effects of TMS over parietal cortex. For instance, Thut et al. (2005) found a general impairment of target detection following leftward cues and an enhancement in the right hemifield following rightward cues after low‐frequency TMS over right PPC. This resembles the general finding of contralateral disruption seen in other experimental paradigms, using line bisection tasks and visual extinction tasks, applying TMS over PPC (line bisection tasks: Brighina et al., 2002; Fierro et al., 2000; Szczepanski & Kastner, 2013; visual extinction tasks: Bien et al., 2012; Cazzoli et al., 2009; Dambeck et al., 2006; Hilgetag et al., 2001; Koch et al., 2005).

There are several potential explanations for the absence of a TMS effect on orienting. Perhaps the selection of the stimulation site based on the international 10–20 EEG positioning system was suboptimal compared to, for example, (f)MRI‐guided localization (Sack et al., 2009), and therefore did not lead to the expected TMS‐induced orienting effects. In the absence of individual fMRI data, we cannot rule out that TMS coil positioning was suboptimal, thus leading to weak or no effects in a subset of participants. However, many previous studies used the 10–20 system, just like we did here and reported positive results. Somewhat surprisingly, we recently even failed to find an effect on orienting after cTBS to right PPC with fMRI‐guided localization (Gallotto et al., 2022).

It thus seems that other factors may be at play. The absence of effects on orienting performance could be explained by the fact that the task required participants to maintain relatively high levels of sustained attention throughout the task. In the current study we used a lateralized flanker‐type task (a small target needs to be differentiated among flankers), whereas in previous studies that have used TMS in investigating functional asymmetries between the left and right hemisphere with regard to spatial attentional control, single lateralized targets were used that had to be detected by participants. Also, compared to Greene et al. (2008) and Asanowicz et al. (2012), our design was more intensely lateralized (target stimuli at 7° eccentricity in our study, 1° in Greene et al., 2008, and 5° in Asanowicz et al., 2012). It is important to note that all these aspects might have put more demands on attentional resources and might have required higher levels of sustained attention (i.e. tonic alertness). Since it has been shown that the alerting system ‘co‐activates’ the parietal cortex involved in spatial orienting (Fernandez‐Duque & Posner, 2001; Posner & Petersen, 1990; Robertson et al., 1998), high levels of voluntary, sustained attention may have eliminated a possible induced orienting deficit. That orienting deficits can be successfully treated with self‐instructional or computerized training methods that focus on improving intrinsic/sustained alertness (Robertson et al., 1995; Sturm et al., 1997; Sturm & Willmes, 2001) further supports the idea that non‐spatial aspects of attentional mechanisms, such as alerting, can have modulatory effects on the orienting system (Chica et al., 2011; Fernandez‐Duque & Posner, 2001; Sturm et al., 2005). It may therefore be the case that orienting performance on the LANT is more robust against TMS modulation.

It is also plausible that the stimulation on the right PPC was not enough to interfere with the task. In previous studies, in general, exogenous orienting tasks (eliciting bottom‐up mechanisms) are used (e.g., Bien et al., 2012), while in this study an endogenous task (with a top‐down component) was used. Given that top‐down orienting is implemented in a bilateral network (Corbetta & Shulman, 2011, 2002), stimulation of the right PPC may not have been enough to interfere with the task. However, for exogenous orienting, which is more right lateralized (Corbetta & Shulman, 2011, 2002), the stimulation of the right PPC would have more consistent effects.

#### 4.2.3 Effects on executive control

As for alerting, there was a significant interaction between brain stimulation and hemifield. We observed a significantly lower cost of conflict in the left compared to the right hemifield in sham TMS, but not in active TMS. Furthermore, regarding the alerting–executive control interaction that was present in the sham data, we found that TMS influenced this relationship in a hemifield specific way. The significant interaction between stimulation, hemifield and cue (neutral vs. no cue) reflected an increased cost of conflict due to active TMS compared to sham TMS in the left compared to the right hemifield (significant for the neutral cue, c.q. alerting cue, trials only). Thus, by applying TMS over right PPC, we found the expected rightward shift of executive control. As for alerting, this conclusion should be read with caution because difference scores did not reach significance in active versus sham TMS, for neither hemifields.

Executive control resolves conflict among competing stimuli (Fan et al., 2002). In the LANT, it is assessed by the flanker task. Although the anterior cingulate cortex and the dorsolateral prefrontal cortex are usually associated with the executive control system (Botvinick et al., 2001; Bush et al., 2000), there is evidence that links parietal cortex to executive control. Firstly, a study found that a patient with bilateral posterior parietal lesions was impaired at filtering out distractors (Friedman‐Hill et al., 2003) suggesting that the PPC plays a role in the top‐down filtering of irrelevant visual information. Furthermore, neuroimaging and patient studies support the theory that several largely non‐overlapping networks ‐ including a fronto‐parietal control network with areas of the PPC – are involved in cognitive control, in which conflict resolution is an essential feature (for review, see Marek & Dosenbach, 2018).

In sum, it is not inconceivable that the behavioural consequences of the stimulation in our study were caused by direct effects by directly hitting the areas of the executive system. Our observed effects on executive control suggest not only a correlational relationship but causality between the PPC and executive control.

### 4.3 Limitation

Our study could be criticized for the specific implementation of the sham stimulation. Instead of a purpose‐built sham TMS coil, we simply tilted the coil by 90° so that the magnetic field was not directed toward the brain. While this approach has been widely used by the TMS community, it can be criticized for multiple reasons. For example, the auditory and somatosensory effects of TMS may not be perfectly matched as the TMS pulse may feel weaker, and the sound may be different. In this context, it seems worthwhile to point out that all control strategies come with their unique disadvantages (Duecker & Sack, 2015b). However, our tilted TMS coil approach is widely accepted as it does mimic sound and sensation (the latter better than a placebo TMS coil). The clicking sound and feeling the weight of the coil on the head of the tilt‐sham approach are known to be well‐matched with active TMS (Duecker & Sack, 2015b). But most importantly, we want to highlight that TMS in our study was not applied during the execution of the task, but rather well before the task started, and therefore we consider it unlikely that our observed behavioural effects after (active or sham) TMS were produced by the clicking or bone‐conducted sound of the TMS coil or sensations on the head. This would be more of a risk when stimulation is given during the execution of a task.

While sham TMS may account for a general placebo (expectation) effect and may also control for direct sensory‐driven behavioural or cognitive changes (clicking and somatosensation), only a control site can test the site‐specificity of our TMS findings and in this sense test how specific these effects are to, for example, parietal cortex. Indeed, in the absence of a control site, no claims can be made about the specific role of PPC and the site‐specificity of our findings. However, this was not the gist of our study. Instead, our study aims to build upon this established and often replicated functional relevance of PPC for attention, now directly comparing three different aspects of attention—orienting, alerting, and executive control. Our main gist is therefore the task‐specificity of these TMS over PPC effects, not site‐specificity. Therefore, we decided for a sham control rather than a control site.

## 5 CONCLUSION

Neuroimaging evidence from previous studies implicated PPC in neural networks sub serving three different types of attention functions: alerting, orienting, and executive control. By assessing them separately one can assess differential effects of TMS‐PPC on these functional components of attention. Our results clearly demonstrate differential brain stimulation effects on two of these components: alerting and executive control. For both these components, TMS over right PPC led to the expected rightward shift (based on the descriptives following the significant interactions). We want to stress here again that the use of flankers made our task clearly different from prior TMS‐PPC studies. This perhaps made orienting performance on the LANT more robust against TMS modulation and may explain why we did not find a direct stimulation effect on orienting. But the demonstrated effects on attention mechanisms of alerting and executive control, rather than the previously revealed role in spatial orienting, emphasize the multifaceted functional contributions of PPC to a range of attention mechanisms. In turn, this implies that future research would benefit from a more inclusive approach, moving from isolated studies of specific aspects of attention to a more integrated approach designed to reveal the intrinsic interplay between attention processes at the behavioural and the neuronal level.

## CONFLICT OF INTEREST

The authors have no relevant financial or non‐financial interests to disclose.

## AUTHOR CONTRIBUTIONS

MMS, FD, SG, CvH, ATS and TS contributed to the conceptualization of the study. MMS led the investigation and was responsible for the data curation. MMS and FD conducted the formal analysis. MMS, ATS and TS wrote the original draft of the manuscript. All authors (MMS, FD, SG, TdG, CvH, ATS and TS) reviewed and edited the manuscript. CvH, ATS and TS provided supervision. ATS was responsible for resources/funding acquisition.

### PEER REVIEW

The peer review history for this article is available at https://publons.com/publon/10.1111/ejn.15830.

## Supporting information


**Data S1.** Supporting Information

## Data Availability

The datasets generated during and/or analysed during the current study are available from the corresponding author on reasonable request.
