# Supplementary material for: Transcranial magnetic stimulation over posterior parietal cortex modulates alerting and executive control processes in attention
Source: Eur J Neurosci. 2022 Oct 12;56(10):5853–68. doi: 10.1111/ejn.15830 (PMC9828423; doi:10.1111/ejn.15830)
Supplement: Supplementary file 1 — Data S1. Supporting Information [file EJN-56-5853-s001.docx]

**Supplementary material**

To be transparent about the greater experimental context of this research report, in this paragraph we describe additional details of the procedure. In both sessions, in addition to (active or sham) TMS, participants received *placebo* transcranial alternating current stimulation (tACS) to left parietal cortex, and brain activity (electroencephalography, EEG) was recorded. TACS and EEG electrodes were attached to the participant’s head using the 10-20 system. Sham tACS was applied to P3 (left PPC) at individual alpha frequency, with intensity set to 1.5 mA peak-to-peak, and consisted of a brief ramping up and immediate ramping down. Sham tACS was delivered during task performance; task duration was between 40-45 minutes. EEG leads were applied to P5, PO3, PO4, P6, left mastoid (A1, online reference), right mastoid (A2, offline reference), and forehead (ground). EEG was recorded three times in each session; before and after (active or sham) TMS and after (sham) tACS (at the end of the session). Participants were required to relax and keep eyes closed during three minutes of EEG recording.
